# Latent Profiles of Motivational and Regulatory Systems and Their Differential Associations with Problematic Gaming and Smoking Behavior Among Chinese College Students: A Cross-Sectional Study

**DOI:** 10.3390/bs16101958

**Published:** 2026-10-21

**Authors:** Jiajia Wu, Peipei Song, Yuxin Gao, Lijin Wang, Haonan Li, Yue Wang, Wanyi Quan, Jing Zhang, Na Zhong, Yonghui Li, Dongliang Jiao

**Affiliations:** 1 School of Mental Health, Bengbu Medical University, Bengbu 233030, China; 2 Shanghai Mental Health Center, School of Medicine, Shanghai Jiao Tong University, Shanghai 200030, China; 3 State Key Laboratory of Cognitive Science and Mental Health, Institute of Psychology, Chinese Academy of Sciences, Beijing 100101, China; 4 Department of Psychology, University of Chinese Academy of Sciences, Beijing 100049, China

**Keywords:** problematic gaming, smoking behavior, latent profile analysis, anxiety, depression, college students

## Abstract

Background: Problematic gaming and smoking are prevalent among college students, yet the extent to which they share or differ in associated psychological profiles remains unclear. This study identified latent psychological profiles based on motivational (Behavioral Inhibition System [BIS] and Behavioral Activation System [BAS]) and regulatory (control/impulse) systems, and examined the associative pathways through which anxiety and depression were linked to the relationships between these profiles and the two addictive behaviors. Methods: This cross-sectional study was conducted between 16 and 29 March 2023. Using convenience sampling, 21 universities were purposively selected from 10 provinces/municipalities in China. A total of 13,643 online questionnaires were returned, and 13,215 valid questionnaires were analyzed after excluding 428 invalid questionnaires. Participants completed measures of BIS/BAS, self-control, problematic gaming, anxiety, depression, smoking behavior, and demographics. Latent profile analysis was used to identify motivational–regulatory profiles. Path analyses estimated indirect associations via anxiety and depression, and moderation analyses examined demographic moderators. All associations were interpreted cross-sectionally. Results: Five distinct profiles were identified: Goal-Oriented (4.44%), Low-Reactive (7.26%), Moderate-Conflict (16.32%), High-Conflict (10.12%), and High-Reactive-Balanced (61.86%). All indirect associations via anxiety and depression were significant for both problematic gaming and smoking. Within problematic gaming, anxiety-related indirect associations were consistently stronger than depression-related ones. Within smoking, anxiety-related and depression-related indirect associations were comparable. Conclusions: These cross-sectional findings indicate that the relative prominence of anxiety- and depression-related pathways may differ within each behavior. Because the two outcomes were measured on different scales, no direct cross-outcome comparison was conducted. Given the cross-sectional design and the measures used, no causal or mechanistic conclusions can be drawn, and the findings should be considered hypothesis-generating.

## 1. Introduction

In the global field of youth health, behavioral addictions and substance use disorders represent two pressing and often overlapping challenges. Notably, problematic gaming and smoking behavior (including conventional tobacco and e-cigarettes) (Song et al., 2023) have emerged as highly prevalent and deleterious conditions among Chinese college students. These behaviors are closely linked to academic burnout, financial strain, and declining physical health, and they frequently co-occur with internalizing disorders such as anxiety and depression (Fluharty et al., 2017; Schou Andreassen et al., 2016). Despite exposure to similar campus environments, individuals show marked variability in their susceptibility to addictive behaviors, suggesting that the configuration of intrinsic psychological traits may serve as a key explanatory factor.

It is important to distinguish between two related but distinct diagnostic terms: ICD-11 includes Gaming Disorder (GD) as a formal diagnosis (van den Brink, 2017) whereas DSM-5 uses Internet Gaming Disorder (IGD) as a condition requiring further research. In the present study, we use the term problematic gaming to refer to elevated symptoms of IGD as assessed by the IGDS9-SF, a self-report screening measure based on DSM-5 criteria; it does not imply a clinical diagnosis of either GD or IGD. Similarly, smoking behavior refers to self-reported use of conventional cigarettes or e-cigarettes at least once in the past 30 days. It is a binary behavioral indicator and does not assess nicotine dependence, smoking frequency, quantity, craving, or withdrawal.

A critical perspective for understanding such variability lies in examining the interaction between motivational drives and regulatory capacity. The Reinforcement Sensitivity Theory, operationalized via the Behavioral Inhibition System/Behavioral Activation System (BIS/BAS) Scale, distinguishes two fundamental neuropsychological systems: the BAS drives reward-approach behavior, while the BIS suppresses behavior in response to punishment cues (Carver & White, 1994). However, motivation alone does not determine behavior. The Dual-Mode Self-Control Model further elaborates the dynamic competition between the control and impulsive systems (Hofmann et al., 2009), capturing an individual’s capacity to regulate impulsive urges. For brevity, we hereafter refer to the BIS/BAS system as the motivational system (encompassing both approach and avoidance motivation) and the Control/Impulse system as the regulatory system.

These two systems map onto two inherently distinct psychological dimensions of addiction vulnerability: motivation dictates whether an individual is susceptible to temptation, and regulation determines whether self-control can be exerted in the face of such temptation. Theoretically, their intersection may generate multiple “motivation–regulation subtypes.” Empirical evidence links specific subtype combinations to addiction: for instance, elevated levels of both BIS and BAS have been observed in addiction-prone populations (Y. Kim et al., 2016). Braddock et al. confirmed that the BAS and BIS jointly modulate impulsivity to influence addiction-related high-risk behaviors, demonstrating that the combinatorial pattern of the motivational and regulatory systems is a robust predictor of addiction vulnerability (Braddock et al., 2011). This complementary relationship is supported by both theory and empirical evidence. From a dual-systems perspective, behavior results from the competition between impulsive/motivational tendencies and reflective/regulatory control (Hofmann et al., 2009). Empirically, Xue et al. (2023) demonstrated that BAS and BIS predict internet addiction through the mediating role of self-control, and Kim-Spoon et al. (2016) showed that inhibitory control moderates the effect of reward sensitivity on substance use. Together, these findings support the integration of motivational and regulatory systems within a single analytical framework.

Compared with single-factor or two-dimensional models, integrating the four core components of the motivational (BIS/BAS) and regulatory (Control/Impulse) systems could more comprehensively explain how motivational activation translates into addictive behaviors under different regulatory conditions. By integrating these indicators in a person-centered analysis, we aim to identify motivational–regulatory profiles that capture the joint influence of approach/avoidance motivation and control/impulse regulation. This approach allows us to examine whether certain combinations of motivational and regulatory characteristics are associated with both behaviors (shared vulnerability) or are specific to one behavior (outcome-specific vulnerability), thereby providing a more comprehensive understanding of the psychological underpinnings of problematic gaming and smoking. However, it remains unknown whether empirically distinct subtypes emerge from the combination of these four factors in real-world populations, and how these hypothetical subtypes differentially relate to addiction risk.

A central question in addiction research concerns the extent to which behavioral addictions (e.g., problematic gaming) and substance-related behaviors (e.g., smoking) share versus differ in their associated psychological vulnerabilities. These possibilities are not mutually exclusive: the two behaviors may partly share broad vulnerability factors, such as altered reward sensitivity and impulse control, while also differing in disorder-specific processes (Sussman & Sinclair, 2025; Zeng et al., 2024). For example, neuroimaging evidence suggests both overlap and divergence in prefrontal and striatal activation patterns between substance-use disorders and behavioral addiction (He et al., 2025). However, it remains unclear to what degree similar psychological profiles—derived from the same set of motivational and regulatory indicators—show convergent versus outcome-specific associations with problematic gaming and smoking behavior within the same population. Person-centered methods can help characterize this degree of convergence and divergence rather than adjudicate between two competing accounts. Negative emotion in addiction constitutes another key research agenda. Koob emphasized that negative reinforcement driven by negative emotion is a core mechanism underlying addiction maintenance (Koob, 2021). According to this model, individuals may engage in addictive behaviors to alleviate withdrawal-induced negative states such as anxiety and depression. However, it remains unclear whether behavioral and substance-use behaviors exhibit identical associations with negative emotion, and how individuals with different psychological profiles differ in these emotional pathways. The present study therefore examines whether anxiety and depression serve as hypothesized indirect associative pathways linking psychological profiles to problematic gaming and smoking behavior.

Moreover, sociodemographic background (e.g., gender, age, household registration, and university type) may moderate the strength of associations between psychological profiles and addictive behaviors (Buono et al., 2020; Costes & Bonnaire, 2022). However, it remains unclear how these factors interact with latent psychological profiles as contextual moderators.

This study proposes an integrated analytical framework to answer two progressive research questions: RQ1: Based on the integrative assessment of motivational (BIS/BAS) and regulatory (control/impulse) systems, what theoretically meaningful latent psychological profiles can be identified among Chinese college students? RQ2: To what extent do these profiles show shared versus outcome-specific associations with problematic gaming and smoking behavior, and to what extent do anxiety and depression account for indirect associations between profile membership and each outcome?

## 2. Materials and Methods

### 2.1. Participants

The survey was conducted online between 16 and 29 March 2023. A total of 13,643 questionnaires were returned. Questionnaires with a completion time of less than 3 min were considered invalid; 428 (3.14%) were excluded, leaving 13,215 valid questionnaires. The valid questionnaire rate among returned questionnaires was 96.86%. The online platform required all items to be answered before submission; therefore, there were no missing data in the retained questionnaires. The platform also allowed only one submission per IP address to prevent duplicate responses.

Convenience sampling was used. Recruitment was conducted through university counseling centers and student affairs offices, which distributed the survey link to students via class WeChat and QQ groups. Inclusion criteria were as follows: (1) currently enrolled full-time undergraduate student at one of the participating universities; (2) aged 18–24 years; and (3) able to read and understand Chinese. No additional participant-level exclusion criteria were applied beyond the invalid questionnaire criteria described above.

Twenty-one universities were selected from 10 provinces/municipalities in China (Anhui, Hunan, Beijing, Jiangsu, Sichuan, Liaoning, Hebei, Guangdong, Guizhou, and Zhejiang). The participating institutions represented diverse institutional orientations, including medical/health (*n* = 2034, 15.4%), science/engineering (*n* = 7765, 58.8%), normal/teacher-training (*n* = 1120, 8.5%), sports (*n* = 1052, 8.0%), public security/judicial (*n* = 458, 3.5%), arts/design (*n* = 495, 3.7%), finance/economics (*n* = 64, 0.5%), comprehensive/applied (*n* = 181, 1.4%), and open-education institutions (*n* = 46, 0.3%). They were located across North (*n* = 1376, 10.4%), East (*n* = 1445, 10.9%), Central (*n* = 8534, 64.6%), South (*n* = 63, 0.5%), Southwest (*n* = 1467, 11.1%), and Northeast (*n* = 330, 2.5%) China. The final sample consisted of 13,215 full-time undergraduate students: freshmen (*n* = 7541, 57.1%), sophomores (*n* = 4436, 33.6%), juniors (*n* = 897, 6.8%), seniors (*n* = 244, 1.8%), and fifth-year students (*n* = 97, 0.7%). This strategy aimed to include universities from regions with diverse economic, geographical, and cultural characteristics; however, it was not designed to be nationally representative.

Convenience sampling may introduce selection bias, because students who voluntarily complete an online survey may differ systematically from the general student population (e.g., in gaming habits, mental health awareness, or willingness to disclose psychological symptoms). This limits the generalizability of the findings to the broader Chinese college student population. Future studies using probability sampling are needed to replicate and extend these findings.

### 2.2. Measures

#### 2.2.1. General Information Questionnaire

This questionnaire collected sociodemographic characteristics including gender, age, type of household registration (urban/rural), grade level, and academic major, as well as information on the use of conventional cigarettes and e-cigarettes. Smoking was assessed by self-report. Conventional cigarette use was defined as ≥1 cigarette in the past 30 days, e-cigarette use as ≥1 use in the past 30 days, and dual use as both. For the main analysis, any use of either product was coded as 1 (past-30-day smoker), and 0 otherwise; this binary indicator was used to assess overall smoking behavior. Product-specific use (conventional only, e-cigarette only, dual use) was also described separately in Section 3.1.

#### 2.2.2. Dual-Mode of Self-Control Scale (DMSC-s)

The Dual-Mode of Self-Control Scale (DMSC-s), with its theoretical framework proposed by Dvorak and Simons in 2009 (Dvorak & Simons, 2009), was used in its Chinese version. This scale consists of 21 items rated on a 5-point Likert scale to measure participants’ self-control capacity. It comprises two systems: the control system and the impulse system. The control system includes two dimensions (future time perspective and problem solving), while the impulse system includes three dimensions (distractibility, low delay gratification, and impulsivity). The score for each dimension is the sum of its corresponding items, with higher scores indicating a higher level of that system. The total self-control score is calculated by summing the control system score and the reverse-scored impulse system score. The scale has demonstrated good reliability and validity. In this study, the Cronbach’s α coefficient for the DMSC-s was 0.915. The Chinese version has shown good reliability and validity in prior research (Xie et al., 2014).

#### 2.2.3. Behavioral Inhibition System/Behavioral Activation System Scale (BIS/BAS Scale)

Participants’ behavioral inhibition and behavioral activation tendencies were assessed using the Behavioral Inhibition System/Behavioral Activation System Scale (BIS/BAS Scale), originally developed by Carver and White (1994). The Chinese version of the scale was used in this study, and its reliability and validity have been supported in previous research (Li et al., 2008). The instrument contains 22 items, four of which are filler items that are not included in the scoring procedure. The remaining items are scored using a 4-point Likert response format. Four components are assessed by the scale: Behavioral Activation System–Drive (BAS-D), Behavioral Activation System–Reward Responsiveness (BAS-R), Behavioral Activation System–Fun Seeking (BAS-F), and Behavioral Inhibition System (BIS). BAS-D, BAS-R, and BAS-F together constitute the Behavioral Activation System (BAS). According to previous psychometric research, the internal consistency coefficient of the total scale was 0.79, the test–retest reliability was 0.80, and the internal consistency coefficients of the individual subscales ranged from 0.52 to 0.73. In the current study, the BIS/BAS Scale yielded a Cronbach’s α coefficient of 0.953. The Cronbach’s α coefficients for each subscale were as follows: BIS (5 items): α = 0.884; BAS Reward Responsiveness (6 items): α = 0.911; BAS Drive (4 items): α = 0.832; BAS Fun Seeking (3 items): α = 0.747.

#### 2.2.4. Patient Health Questionnaire-9 (PHQ-9)

The Patient Health Questionnaire-9 (PHQ-9) is a 9-item self-report measure assessing the frequency and extent to which individuals have been bothered by problems related to depression, such as low mood and loss of interest, over the past two weeks (Kroenke et al., 2001). Items are rated on a 4-point Likert scale, with total scores ranging from 0 to 27. Scores of 0–4 indicate no depression; 5–9 indicate possible mild depression; 10–14 indicate possible moderate depression; 15–19 indicate possible moderately severe depression; and 20–27 indicate possible severe depression. Higher scores reflect more severe depressive symptoms. In this study, the Cronbach’s α coefficient for the PHQ-9 was 0.923. The Chinese version has shown good reliability and validity in prior research (W. Wang et al., 2014).

#### 2.2.5. Generalized Anxiety Disorder-7 (GAD-7)

The Generalized Anxiety Disorder-7 (GAD-7) is a 7-item self-report measure assessing the frequency and extent to which individuals have been bothered by problems related to anxiety, such as feeling nervous or worried, over the past two weeks (Spitzer et al., 2006). Items are rated on a 4-point Likert scale, with total scores ranging from 0 to 21. Scores of 0–4 indicate no anxiety; 5–9 indicate possible mild anxiety; 10–13 indicate possible moderate anxiety; 14–18 indicate possible moderately severe anxiety; and 19–21 indicate possible severe anxiety. Higher scores reflect more severe anxiety symptoms. In this study, the Cronbach’s α coefficient for the GAD-7 was 0.943. The Chinese version has shown good reliability and validity in prior research (Y. Wang et al., 2018).

#### 2.2.6. Internet Gaming Disorder Scale–Short-Form (IGDS9-SF)

The Internet Gaming Disorder Scale–Short-Form (IGDS9-SF), developed by Pontes and Griffiths (2015), was used to assess symptoms related to Internet Gaming Disorder. The scale assesses problematic gaming symptoms over the past 12 months. The Chinese version was adopted in this study and has demonstrated good psychometric properties in previous research; it has proposed a score of 32 as a screening cut-off for identifying individuals with elevated IGD symptoms (Qin et al., 2020). The IGDS9-SF was developed with reference to the nine diagnostic criteria for Internet Gaming Disorder described in the fifth edition of the Diagnostic and Statistical Manual of Mental Disorders (DSM-5). It contains nine items, with responses recorded on a 5-point Likert scale ranging from 1 (“Never”) to 5 (“Very frequently”). The summed score can range from 9 to 45, and higher scores indicate a greater severity of IGD-related symptoms. The Cronbach’s α coefficient obtained for the IGDS9-SF in the present study was 0.940.

### 2.3. Data Analysis

Descriptive and correlation analyses were conducted using SPSS version 27.0. Descriptive statistics were computed for all study variables. Continuous variables were summarized as means and standard deviations (M ± SD), including age, IGDS9-SF score, GAD-7 score, PHQ-9 score, BIS, BAS, control system, and impulse system. Categorical variables were summarized as frequencies and percentages (*n*, %), including gender, residence, type of university, smoking status, and the proportion of participants with an IGDS9-SF score ≥32. Pearson correlation analyses were used to examine the relationships among continuous variables, including BAS, BIS, control system, impulse system, IGDS9-SF, GAD-7, and PHQ-9. For the binary variable smoking status (0 = non-smoker, 1 = smoker), point-biserial correlations were computed between smoking status and the continuous variables. All correlation analyses were two-tailed, and statistical significance was set at *p* < 0.05.

Mplus software (version 8.3) was utilized for the following analyses:

(1) Latent Profile Analysis (LPA) was conducted using the Behavioral Inhibition System (BIS), Behavioral Activation System (BAS), and the control and impulse systems from the self-control scale to identify latent subtypes within the population. Latent profile analysis (LPA) was conducted in Mplus 8.3 using four continuous indicators: BAS, BIS, Control system and Impulse system. Missing data were coded as -999; no missing data were present on these indicators (N = 13,215). Models were estimated using robust maximum likelihood (MLR). We specified class-invariant within-class variances and fixed within-class covariances to zero (i.e., local independence). For each model, we used 500 random starting values and retained the 100 best for final-stage optimization. Convergence was assessed by normal termination and replication of the best log-likelihood across random starts. We compared solutions with 1 to 6 profiles using AIC, BIC, sample-size-adjusted BIC, the Vuong–Lo–Mendell–Rubin likelihood ratio test (VLMR-LRT), the Lo–Mendell–Rubin adjusted LRT, the parametric bootstrapped likelihood ratio test (BLRT), entropy, class proportions, and interpretability.

(2) Path analysis was conducted to examine the indirect associations of anxiety and depression in the relationships between the latent profiles and the two addictive outcomes. The latent class variable (dummy-coded, with Profile 5 as reference) was specified as the independent variable, anxiety (GAD-7) and depression (PHQ-9) as parallel pathway variables, and problematic gaming and smoking behavior as outcomes. The residual covariance between GAD-7 and PHQ-9 was constrained to zero. Therefore, the model was not saturated. Because of the cross-sectional nature of the data, all estimates are interpreted as associations, not causal effects. Bias-corrected bootstrap confidence intervals based on 5000 resamples were used for the indirect effects and their contrasts.

The measures used in this study had different recall periods: anxiety and depression were assessed over the past two weeks, problematic gaming over the past 12 months, and smoking over the past 30 days. This temporal mismatch limits the interpretation of the indirect associations and precludes strong conclusions about temporal ordering.

(3) Moderation analyses were conducted to examine whether demographic variables moderated the associations between profiles and outcomes. No adjustment for multiple testing was applied. Given the number of interaction tests, these analyses were considered exploratory, and uncorrected *p* values should be interpreted with caution.

For all statistical analyses, * *p* < 0.05 was considered indicative of a statistically significant difference.

## 3. Results

### 3.1. General Demographic Characteristics

The demographic characteristics of the study sample are presented in Table 1. In this study, “having smoked at least once in the past 30 days” was used as the operational definition of “current smokers” among college students (Kasza et al., 2020). A total of 13.5% of college students reported smoking behavior. Notably, the proportion of students reporting current smoking among male students (19.45%) was significantly higher than that among female students (3.74%) (χ^2^ = 658.85, *p* < 0.001). Among all participants, 1782 (13.5%) reported any past-30-day smoking. Specifically, 1462 (11.07%) reported conventional cigarette use only, 56 (0.43%) reported e-cigarette use only, and 264 (2.00%) reported dual use. Furthermore, 3.12% of students screened positive for problematic gaming (IGDS9-SF score ≥ 32). Regarding the comorbidity of smoking and problematic gaming, 84.3% of the students had neither, 12.6% only smoked, 2.2% only had problematic gaming, and 0.9% had both smoking and problematic gaming, resulting in a combined smoking rate of 13.5% and a problematic gaming positivity rate of 3.1%.

### 3.2. Correlation Analysis

Examining the correlations among the main study variables serves to provide a foundation for subsequent profile characterization and mediation pathway analyses. As shown in Table 2, IGDS9-SF scores were positively correlated with BIS (r = 0.137), the Impulse System (r = 0.388), GAD-7 (r = 0.565), PHQ-9 (r = 0.368), and smoking behavior (r = 0.089). The association between IGDS9-SF and the Control System was not statistically significant (r = −0.013). Anxiety and depression showed similar correlation patterns, both positively associated with BIS, the impulse system, and the two addictive behaviors, while negatively associated with the control system. Smoking behavior was weakly but significantly correlated with most variables except the impulse system.

### 3.3. Profile Analysis

Latent profile analysis (LPA) was conducted to identify subtypes based on the motivational (BIS/BAS) and regulatory (control/impulse) systems. Fit indices for 2- to 6-profile models are presented in Table 3. Although the 6-profile model yielded the lowest AIC and highest entropy, it contained a class with a very small proportion (2%), raising concerns about interpretability and stability. The five-profile solution had all class proportions above 4% and each profile was clearly interpretable. The Lo–Mendell–Rubin test indicated that the 5-profile model fit significantly better than the 4-profile model (*p* < 0.001). We note that the 6-profile solution showed evidence of instability. Although the best log-likelihood was replicated across random starts, the parametric bootstrapped LRT produced only 5 successful bootstrap draws, and 3 of these draws had smaller LRT values and non-replicated best log-likelihoods for the 6-profile model. Mplus warned that the *p*-value may not be trustworthy due to local maxima and recommended increasing the number of random starts using the LRTSTARTS option. Given this instability, the 6-profile solution was not retained as the final model. Based on model fit indices and theoretical interpretability, the 5-profile solution was retained. Detailed information regarding the 6-profile analysis can be found in the Supplementary Materials.

The 5-profile model showed good classification accuracy (Table 4), with average posterior probabilities ranging from 0.839 to 0.985, indicating reliable class assignment.

**Figure 1:**
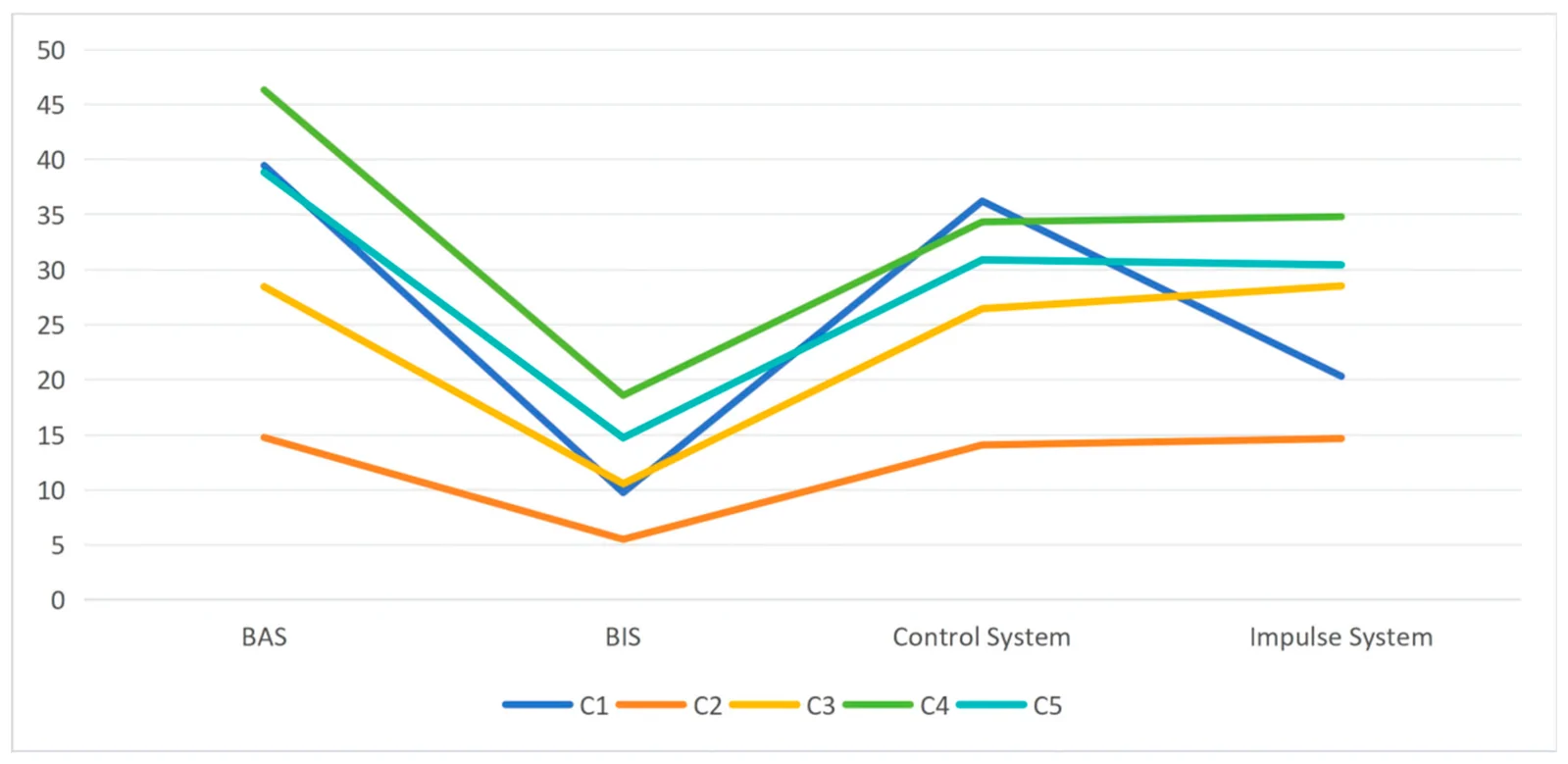
Mean score patterns of the five latent profiles. Note. BAS = Behavioral Activation System; BIS = Behavioral Inhibition System.

Table 5 presents the characteristics of the five profiles and Figure 1 illustrates the mean score patterns. The five profiles were labeled as follows:

C1 (Goal-Oriented, 4.44%): High BAS, high control, low BIS, and low impulse.

C2 (Low-Reactive, 7.26%): Lowest scores across all four systems.

C3 (Moderate-Conflict, 16.32%): Moderate scores across all systems.

C4 (High-Conflict, 10.12%): Highest scores across all systems.

C5 (High-Reactive-Balanced, 61.86%): Relatively high and balanced scores across all systems.

The BCH (Bolck–Croon–Hagenaars) tests showed that the five profiles differed significantly in IGDS9-SF, GAD-7, PHQ-9, and smoking behavior (all *p* < 0.001; see Table 6).

### 3.4. Path Analysis of Indirect Associations

Parallel mediation analysis was conducted using Profile 5 (High-Reactive-Balanced) as the reference category, with anxiety (GAD-7) and depression (PHQ-9) as parallel mediators. LPA1–LPA4 were included as dummy variables. Problematic gaming was treated as a continuous outcome and modeled using linear regression; smoking behavior was treated as a binary outcome and modeled using logistic regression. Indirect effects were defined as the product of the corresponding a and b paths (a × b). To formally test whether the anxiety- and depression-specific indirect associations differed in magnitude, we computed the contrast between the GAD-7-specific and PHQ-9-specific indirect effects within each profile comparison (LPA1 vs. Profile 5, LPA2 vs. Profile 5, LPA3 vs. Profile 5, and LPA4 vs. Profile 5), separately for problematic gaming and smoking behavior. The 95% confidence intervals (CIs) for these contrasts were obtained using bias-corrected bootstrap with 5000 draws. All models controlled for age, gender, household registration, university type, and grade. The residual covariance between GAD-7 and PHQ-9 was constrained to zero; therefore, the model was not saturated (for problematic gaming, df = 1). Results are presented in Table 7 and Table 8.

For problematic gaming, the Goal-Oriented (LPA1) and Low-Reactive (LPA2) profiles showed significant negative indirect effects via both anxiety and depression, whereas the Moderate-Conflict (LPA3) and High-Conflict (LPA4) profiles showed significant positive indirect associations via both mediators. All indirect effects had 95% CIs excluding zero. The contrasts between the anxiety- and depression-specific indirect effects were significant for all profile comparisons: LPA1 vs. Profile 5, −2.274 (95% CI [−2.506, −2.048]); LPA2 vs. Profile 5, −1.074 ([−1.332, −0.823]); LPA3 vs. Profile 5, 1.418 ([1.217, 1.632]); LPA4 vs. Profile 5, 1.700 ([1.418, 2.013]). These results indicate that the anxiety-specific indirect association was significantly stronger than the depression-specific indirect association for problematic gaming across all profile comparisons.

For smoking behavior, LPA1 and LPA2 showed negative indirect effects via both mediators, while LPA3 and LPA4 showed positive indirect effects. The contrast between the anxiety- and depression-specific indirect effects was significant only for LPA2 vs. Profile 5 (D = 0.111, 95% CI [0.054, 0.165]), indicating that depression contributed more strongly than anxiety for this profile comparison. For the remaining comparisons, the contrasts were not significant: LPA1 vs. Profile 5, 0.050 ([−0.018, 0.119]); LPA3 vs. Profile 5, 0.020 ([−0.015, 0.051]); LPA4 vs. Profile 5, −0.028 ([−0.081, 0.022]). Thus, for smoking behavior, the anxiety and depression pathways did not differ significantly in most profile comparisons.

Comparisons of indirect effects across problematic gaming and smoking were not performed because the two outcomes were modeled on different statistical scales (continuous vs. binary).

### 3.5. Moderation Effect Test

Moderation analyses. Using game score and smoking status as dependent variables and profile type as the independent variable, a series of moderation analyses were conducted, with gender, household registration type, university type, and age as moderating variables. Four dummy-coded LPA variables (LPA1–LPA4) were created, with LPA5 as the reference category. Thus, all LPA effects represent differences relative to LPA5. Gender was coded as [0 = male, 1 = female]; household registration type was coded as [0 = urban, 1 = rural]; university type was coded as [0 = general university, 1 = advanced university]. Age was treated as continuous and grand-mean-centered before creating interaction terms.

For each moderator, product terms were created between the moderator and each LPA dummy variable: LPA1 × Moderator, LPA2 × Moderator, LPA3 × Moderator, and LPA4 × Moderator. Separate hierarchical regression models were estimated for each moderator. In Step 1, the main-effects model included LPA1–LPA4 and all demographic variables (gender, age, household registration type, and university type). In Step 2, the set of interaction terms between LPA1–LPA4 and the focal moderator was added.

As shown in Table 9, gender significantly moderated the relationships between psychological profiles and both problematic gaming (ΔR^2^ = 0.003, *p* < 0.001) and smoking behavior (Δχ^2^(4) = 24.890, *p* < 0.001). Age significantly moderated the profile–problematic gaming relationship (ΔR^2^ = 0.002, *p* < 0.001) but not the profile–smoking relationship (*p* = 0.500). Household registration type showed no moderating effects on either outcome. For smoking behavior, the overall interaction between university type and profiles was not significant (*p* = 0.062). The class-specific coefficient for the Moderate-Conflict profile (OR = 0.753, *p* = 0.047) is reported for transparency only and should be treated as exploratory. It should not be interpreted as evidence of moderation.

## 4. Discussion

Through latent profile analysis of 13,215 college students, this study identified five psychological profiles based on the Behavioral Inhibition/Activation System (BIS/BAS) and the self-control system (control/impulse). The study further examined the relationships between different psychological profiles and problematic gaming as well as smoking behavior, explored indirect associations via anxiety and depression and the moderating effects of demographic variables. Among Chinese college students in this sample, the results indicate that problematic gaming and smoking behavior showed different patterns of association with psychological profiles, emotional pathways, and demographic moderators. These cross-sectional findings suggest differential association patterns.

### 4.1. Characteristics of the Psychological Profiles and Their Associations with Addictive Behaviors

This study identified five psychological profiles: Goal-Oriented (4.44%), Low-Reactive (7.26%), Moderate-Conflict (16.32%), High-Conflict (10.12%), and High-Reactive-Balanced (61.86%).

C1 (Goal-Oriented) was characterized by high approach motivation, high control, low inhibition, and low impulsivity. This profile showed the lowest scores for anxiety, depression, and problematic gaming. However, its prevalence of past-30-day smoking (16.9%) was higher than that observed in C4 and C5. Therefore, C1 may be favorable with respect to emotional symptoms and problematic gaming, but not with respect to smoking behavior.

C2 (Low-Reactive) showed the lowest scores across all four systems and had the highest prevalence of past-30-day smoking (23.0%), while its anxiety and depression levels were relatively low. Because smoking was assessed only as any past-30-day smoking, this binary measure does not capture nicotine dependence, smoking motives, frequency, or quantity and may include social or occasional smoking. Therefore, the reasons for the higher smoking prevalence in C2 cannot be determined from the present data. Previous research has suggested that some substance use behaviors may be associated with anhedonia or low arousal (Garfield et al., 2014), and that social or sensory motives may also play a role in smoking and e-cigarette use (Lindpere et al., 2023). However, because these constructs were not measured in this study, these interpretations remain speculative. Future studies with detailed smoking measures are needed to clarify this pattern.

C3 (Moderate-Conflict) and C4 (High-Conflict) showed higher scores across the BAS, BIS, control, and impulse systems. College students in these profiles reported both stronger reward-approach tendencies (high BAS) and heightened threat sensitivity (high BIS); despite relatively high control scores, their elevated impulsivity may reduce the effectiveness of self-regulation. C3/C4 and C1 all showed relatively high BAS and control scores, but C3/C4 had higher BIS, impulsivity, anxiety, depression, and problematic gaming than C1. Among Chinese college students in this sample, one hypothesis is that similar levels of self-reported control may function differently depending on motivational context: control may be more effective in C1 (high BAS, low BIS) but less effective in C3/C4 (high BAS, high BIS), a pattern that could be described as “ineffective overcontrol” (Gilbert et al., 2020). However, this interpretation is speculative, and no neurobiological data were collected in this study. Future studies with neural or behavioral measures of self-regulation are needed to test whether this pattern reflects compensatory or ineffective control processes (He et al., 2025).

C5 (High-Reactive-balanced) represented one of the healthier subgroups in this study, exhibiting the lowest smoking rate, with problematic gaming and emotional symptoms at moderately low levels. Notably, C5 had intermediate scores on all motivation–regulation factors (between C3 and C4) but significantly lower levels of anxiety and depression, and a correspondingly lower gaming addiction score. This pattern is consistent with the indirect association via anxiety observed in the path model: the lower vulnerability to problematic gaming in C5 may be less attributable to its “intermediate” motivation–regulation traits than to its lower levels of negative emotion. However, given the cross-sectional design, this pattern should be interpreted descriptively, and causal interpretations are not warranted.

The four-factor integration allowed us to describe profiles based on both motivational and regulatory indicators. However, BIS and BAS were highly correlated (r = 0.795), raising the possibility that the profile solution partly reflects a general dimension of psychological reactivity rather than four clearly separable systems. Therefore, the present findings should be interpreted as tentative and partial support for the integrative framework. Future research should examine the discriminant validity of the four systems and test whether these profiles replicate with independent measures.

### 4.2. Differences in Emotional Association Patterns: Anxiety Shows Stronger Indirect Associations with Problematic Gaming than with Smoking

Before examining the differences, it is worth noting that anxiety and depression showed significant indirect associations between psychological profiles and both problematic gaming and smoking, as all indirect effects were statistically significant (with 95% confidence intervals excluding zero; see Table 7 and Table 8). This indicates that negative emotions play a non-negligible role in both addictive behaviors. However, the magnitude of these indirect associations differed markedly between the two outcomes.

For problematic gaming, the contrasts between the anxiety- and depression-specific indirect associations were significant across all profile comparisons (see Table 7), indicating that the anxiety-related indirect association was consistently stronger than the depression-related one. For smoking behavior, most of these contrasts were not significant, except for LPA2 vs. Profile 5, where the depression-related indirect association was stronger. These patterns suggest that anxiety-related pathways may be more consistently involved in problematic gaming than in smoking. However, the two outcomes were assessed on different measurement scales: problematic gaming was measured as a continuous nine-item symptom-severity score, whereas smoking was a binary indicator. Because of this asymmetry, the magnitudes of indirect associations across the two outcomes were not directly compared, and the present findings do not establish that anxiety is the “key emotional driver” of problematic gaming or that emotional processes are secondary in smoking.

These findings align with the self-medication theory (Khantzian, 1997), which proposes that individuals may engage in addictive behaviors to alleviate negative emotional states. Anxiety, as a core emotional experience of approach–avoidance conflict, may be one candidate emotional pathway associated with problematic gaming (D. J. Kim et al., 2017). However, because the data are cross-sectional and emotion regulation, gaming motives, craving, and withdrawal were not measured, this interpretation remains hypothesis-generating.

For smoking behavior, the indirect associations via anxiety and depression were statistically significant in most profile comparisons, but their magnitudes were generally comparable, and no consistent anxiety-dominant pattern emerged. This pattern does not indicate that emotional processes are secondary in smoking, nor does it identify non-emotional factors as primary drivers. Nicotine pharmacology, habit, and social context are plausible candidates, but they were not measured in this study and should be examined in future research.

Chinese college students often study and live in highly competitive campus environments, with strong family expectations and cultural norms that may influence mental health help-seeking (Khan et al., 2026). In this setting, anxiety and depression may be expressed through internalizing symptoms, and gaming can serve as an accessible way to escape stress or regulate negative emotions (Hu et al., 2025). This may partly explain why problematic gaming showed a more consistent anxiety-related association in our sample. On the other hand, smoking among Chinese young adults—especially men—is often socially embedded, and e-cigarette use has become increasingly visible among urban youth (Sheer & Mao, 2017; Wu et al., 2025). These behaviors may be maintained more by peer context, social norms, and product accessibility than by emotional distress, which is consistent with the weaker emotional associations for smoking (Zhou et al., 2023). Because cultural norms, health behaviors, and socioeconomic conditions were not directly measured, these interpretations remain hypothesis-generating and should be tested in future research.

Taken together, in this sample of Chinese college students, the two behaviors showed both convergent and outcome-specific association patterns. Both were associated with emotional pathways, but the pattern was more consistently anxiety-related for problematic gaming. These cross-sectional patterns should be interpreted descriptively and as hypothesis-generating, and they do not establish dissociable psychological mechanisms.

Given the cross-sectional design, the indirect associations reported here do not demonstrate causal mediation. Alternative directional explanations are equally plausible—for example, problematic gaming symptoms may increase anxiety and depression, which in turn may alter psychological trait expressions. The path model should be understood as a statistical decomposition of associations that is consistent with the hypothesized temporal order, but not as evidence of that order. Readers are cautioned against causal interpretations, and future research should employ longitudinal or experimental designs to test the directional hypotheses suggested by these patterns.

### 4.3. Moderation by Demographic Variables: Small but Detectable Effects

Moderation analyses showed that gender and age had statistically detectable but small moderation effects on the profile–problematic gaming relationship (ΔR^2^ = 0.003 for gender and 0.002 for age, both *p* < 0.001). These effect sizes indicate that demographic variables explained only a very small proportion of additional variance. Given the large sample size, these effects should be interpreted as statistically detectable but of limited practical significance. The age-related moderation cannot be interpreted as evidence of developmental change or natural remission because the data are cross-sectional.

For smoking behavior, the overall interaction between university type and profiles was not significant (*p* = 0.062). The class-specific coefficient for the Moderate-Conflict profile (OR = 0.753, *p* = 0.047) should therefore be treated as exploratory and not overinterpreted. Smoking was measured only as any past-30-day smoking and does not assess nicotine dependence; therefore, these findings should not be described as reflecting dependence-related processes. Overall, demographic moderation effects in this study were small and should not be emphasized as major findings.

### 4.4. Theoretical Integration: Convergence and Divergence in Association Patterns

Synthesizing the above findings, this study observed convergent and outcome-specific association patterns.

Problematic gaming showed a pattern of associations that is consistent with, but does not demonstrate, an emotion-regulation account. In this study, problematic gaming was more consistently linked to anxiety-related indirect associations, and some profiles with higher BAS/BIS scores also showed higher problematic gaming scores. However, emotion regulation, gaming motives, craving, and withdrawal were not measured; therefore, these interpretations remain hypothesis-generating.

For smoking behavior, the indirect associations via anxiety and depression were statistically significant in most profile comparisons, but they were comparable in magnitude, and no consistent anxiety-dominant pattern emerged. This pattern may be consistent with non-emotional factors such as nicotine pharmacology, conditioned reinforcement, or social context, but these constructs were not assessed. Because smoking was measured only as any past-30-day smoking, these findings should not be interpreted as reflecting nicotine dependence.

Taken together, the two behaviors showed both convergent and outcome-specific association patterns. Both were associated with emotional pathways, but the pattern was more consistently anxiety-related for problematic gaming. Given the cross-sectional design and different outcome measures, these patterns should be interpreted descriptively and as hypothesis-generating.

Future research should include measures of emotion regulation, gaming motives, craving, and withdrawal for problematic gaming, and measures of smoking motives, nicotine dependence, craving, withdrawal, conditioned reinforcement, and habitual behavior for smoking. Longitudinal designs with comparable measures across both outcomes would help test whether these candidate mechanisms account for the observed association patterns.

### 4.5. Practical Implications

The present findings may have potential implications for practice, but they should be interpreted cautiously given the cross-sectional design. The observed association patterns suggest that emotion-regulation-related factors may be relevant for problematic gaming, whereas habit-related or contextual factors may be relevant for smoking. These patterns could inform the development of future interventions. However, this study did not evaluate any intervention, and no evidence-based recommendations can be made. Gender-specific approaches may also warrant consideration in future research, but the current findings are hypothesis-generating and require replication in longitudinal and intervention studies before practical guidelines can be derived.

### 4.6. Limitations

This study has several limitations. First, the sampling strategy was not probabilistic: 21 universities were purposively selected, and participants were recruited by convenience sampling. Although the participating universities were geographically and institutionally diverse, the sample was not designed to be nationally representative, and findings should be generalized with caution. Second, all measures were self-reported, which may introduce recall bias, social desirability bias, and misclassification. The online survey format may also have led to response bias, including inattentive or inconsistent responding. Third, the two outcomes were measured on different scales and are not equivalent. Problematic gaming was assessed with a continuous nine-item symptom-severity measure, whereas smoking was reduced to a binary indicator of any past-30-day use. Therefore, this study did not compare two disorders or two measures of addiction severity, and the magnitudes of associations across the two outcomes should not be directly compared. Fourth, conventional cigarette use and e-cigarette use were combined into a single binary smoking indicator. These forms of nicotine use may represent partly different phenomena with different social and motivational correlates, and combining them may obscure product-specific patterns. In addition, this binary measure does not capture smoking frequency, quantity, motives, or nicotine dependence. Fifth, the cross-sectional design precludes causal or mechanistic inferences. The temporal ordering of profiles, emotional pathways, and behavioral outcomes cannot be established, and the indirect associations should be interpreted as descriptive and hypothesis-generating. Sixth, several constructs relevant to the proposed interpretations were not measured, including emotion regulation, gaming motives, craving, withdrawal, smoking motives, nicotine dependence, conditioned reinforcement, and habitual behavior. Future research with these measures is needed to test the candidate mechanisms suggested by the present findings. Finally, the sample was limited to Chinese college students. Although the proportions of smoking-only, problematic gaming-only, and co-occurring groups were described in Section 3.1, these subgroups were not examined in the inferential analyses. Future research should examine these subgroups and test whether the findings replicate in other populations and with independent measures.

## 5. Conclusions

In this sample of Chinese college students, latent profile analysis identified five motivational–regulatory profiles based on BIS/BAS and self-control systems. Anxiety and depression showed significant indirect associations with both problematic gaming and smoking behavior. For problematic gaming, the anxiety-related indirect association was consistently stronger than the depression-related one, whereas for smoking behavior, the two pathways were generally comparable. Demographic moderation effects were statistically detectable but small. These cross-sectional findings suggest differential association patterns for problematic gaming and smoking behavior, but they do not establish causal mechanisms. Because problematic gaming was assessed with a screening measure and smoking was defined as any past-30-day use, no clinical or dependence-related conclusions can be drawn. The findings should be considered hypothesis-generating and require replication in longitudinal studies with more detailed measures.

## Figures and Tables

**Table 1:** General Information Data (N = 13,215).

Variables	Number (%)/(M ± SD)
Gender	
Male	8193 (62%)
Female	5022 (38%)
Residence	
Rural	8710 (65.9%)
City	4505 (34.1%)
Type of university	
General institutions of higher education	5531 (41.85%)
Advanced vocational school	7684 (58.15%)
Grade	
Freshman	7541 (57.1%)
Sophomore	4436 (33.6%)
Junior	897 (6.8%)
Senior	244 (1.8%)
Junior-year	97 (0.7%)
Smoking/problematic gaming co-occurrence	
Neither	11,138 (84.3%)
Smoking only	1664 (12.6%)
Problematic gaming only	295 (2.2%)
Both	118 (0.9%)
Smoker	1782 (13.5%)
Internet Gaming	
IGDS9-SF score	17.07 ± 7.29
IGDS9-SF score ≥ 32	413 (3.12%)
Age	19.46 ± 1.26
GAD-7	3.73 ± 4.46
PHQ-9	4.60 ± 5.15
BIS	13.54 ± 3.51
BAS	36.18 ± 8.51
Control systems	29.53 ± 7.68
Impulse system	28.97 ± 9.10

Note. Data are presented as *n* (%) or M ± SD. Smoking was defined as having smoked at least once in the past 30 days; IGDS9-SF = Internet Gaming Disorder Scale–Short-Form; GAD-7 = Generalized Anxiety Disorder-7; PHQ-9 = Patient Health Questionnaire-9; BAS = Behavioral Activation System; BIS = Behavioral Inhibition System.

**Table 2:** Correlations among study variables.

	BAS	BIS	Control System	Impulse System	IGDS9-SF	GAD-7	PHQ-9	(Smoker/Non-Smoker)
BAS	1							
BIS	0.795 **	1						
Control System	0.611 **	0.471 **	1					
Impulse System	0.382 **	0.487 **	0.238 **	1				
IGDS9-SF	0.042 **	0.137 **	−0.013	0.388 **	1			
GAD-7	0.002	0.144 **	−0.026 **	0.375 **	0.565 **	1		
PHQ-9	0.111 **	0.232 **	0.036 **	0.376 **	0.368 **	0.614 **	1	
Smoker/Non-smoker	−0.086 **	−0.099 **	−0.071 **	0.001	0.089 **	0.105 **	0.100 **	1

Note. BAS = Behavioral Activation System; BIS = Behavioral Inhibition System; IGDS9-SF = Internet Gaming Disorder Scale–Short-Form; GAD-7 = Generalized Anxiety Disorder-7; PHQ-9 = Patient Health Questionnaire-9. Smoking behavior was coded as 0 = non-smoker, 1 = smoker. ** *p* < 0.01.

**Table 3:** Comparison of fit indices for latent profile models (N = 13,215).

Profiles	AIC	BIC	aBIC	Entropy	LMR (*p*)	BLRT (*p*)	Class Proportions
2	333,414.44	333,511.79	333,470.48	0.963	<0.001	<0.001	0.11/0.89
3	327,826.02	327,960.82	327,903.62	0.894	<0.001	<0.001	0.07/0.19/0.74
4	324,229.17	324,401.42	324,328.33	0.894	<0.001	<0.001	0.67/0.17/0.07/0.09
5	322,167.92	322,377.61	322,288.63	0.903	<0.001	<0.001	0.04/ 0.07/0.16/0.11/0.62
6	320,876.32	321,123.46	321,018.59	0.909	<0.001	<0.001	0.04/0.02/0.16/0.06/0.61/0.11

Note: AIC = Akaike Information Criterion; BIC = Bayesian Information Criterion; aBIC = sample-size adjusted BIC; LMR = Lo–Mendell–Rubin test; BLRT = Bootstrap Likelihood Ratio Test.

**Table 4:** Average posterior probabilities for the 5-class latent profile model (row = actual class, column = predicted class).

	C1	C2	C3	C4	C5
C1	0.839	0.000	0.062	0.000	0.099
C2	0.000	0.985	0.015	0.000	0.000
C3	0.023	0.009	0.912	0.000	0.056
C4	0.000	0.000	0.000	0.898	0.102
C5	0.010	0.000	0.017	0.022	0.950

**Table 5:** Comparison of latent profile characteristics.

Class	BAS	BIS	Control System	Impulse System	Proportion	Profile Characteristic Description
C1	39.457	9.746	36.220	20.314	4.44%	High activation + High control Low inhibition + Low impulse
C2	14.745	5.501	14.074	14.652	7.26%	Low across all systems
C3	28.440	10.554	26.455	28.522	16.32%	Moderate and balanced
C4	46.315	18.572	34.332	34.813	10.12%	High across all systems
C5	38.837	14.722	30.886	30.427	61.86%	Relatively high and balanced

Note. BAS = Behavioral Activation System; BIS = Behavioral Inhibition System. Values represent mean scores for each system across the five identified profiles. Profile labels are based on the pattern of scores across the four systems. Proportions indicate the percentage of the total sample (N = 13,215) assigned to each profile.

**Table 6:** Comparison of latent profile scores on external validity indicators (BCH method) (M ± SE)/[*n*(%)].

Variable	C1	C2	C3	C4	C5	χ^2^ (*p*)
GAD-7	0.02 ± 0.11 ^a^	2.04 ± 0.13 ^b^	5.40 ± 0.12 ^c^	5.79 ± 0.18 ^c^	3.41 ± 0.05 ^d^	1474.836 ***
PHQ-9	0.98 ± 0.15 ^a^	1.21 ± 0.10 ^a^	5.53 ± 0.13 ^b^	6.99 ± 0.22 ^c^	4.63 ± 0.06 ^d^	1683.784 ***
IGDS9-SF	11.26 ± 0.25 ^a^	12.86 ± 0.22 ^b^	19.56 ± 0.18 ^c^	18.47 ± 0.29 ^d^	17.10 ± 0.08 ^e^	1058.652 ***
Smoking	91(16.9%) ^a^	220(23.0%) ^b^	418(19.3)% ^a^	171(13.3%) ^c^	882(10.6%) ^d^	141.644 ***

Note. Data are presented as mean ± standard error (M ± SE) for continuous variables (GAD-7, PHQ-9, IGDS9-SF) and as percentage [*n*(%)] for smoking behavior. BCH = Bolck–Croon–Hagenaars method. Post-hoc comparisons were performed using pairwise Wald tests. GAD-7 = Generalized Anxiety Disorder-7; PHQ-9 = Patient Health Questionnaire-9; IGDS9-SF = Internet Gaming Disorder Scale–Short-Form. C1 = Goal-Oriented; C2 = Low-Reactive; C3 = Moderate-Conflict; C4 = High-Conflict; C5 = High-Reactive-Balanced. *** *p* < 0.001. Within each row, values sharing the same superscript letter do not differ significantly at *p* < 0.05. For smoking behavior, percentages are based on the following class denominators: C1 *n* = 536, C2 *n* = 955, C3 *n* = 2153, C4 *n* = 1287, C5 *n* = 8284.

**Table 7:** Parallel mediation effects of latent profiles on problematic gaming and smoking through anxiety and depression.

Profile Comparison	GAD-7 Indirect	95% CI	PHQ-9 Indirect	95% CI	GAD − PHQ Contrast	95% CI
Dependent variable: IGDS9-SF						
Profile1 vs. Profile 5	−2.385	[−2.570, −2.187]	−0.110	[−0.202, −0.022]	−2.274	[−2.506, −2.048]
Profil2 vs. Profile 5	−1.196	[−1.418, −0.963]	−0.122	[−0.223, −0.025]	−1.074	[−1.332, −0.823]
Profil3 vs. Profile 5	1.446	[1.242, 1.658]	0.028	[0.007, 0.056]	1.418	[1.217, 1.632]
Profil4 vs. Profile 5	1.775	[1.488, 2.079]	0.075	[0.015, 0.143]	1.700	[1.418, 2.013]
Dependent variable: Smoker/Non-smoker						
Profil1 vs. Profile 5	−0.092	[−0.132, −0.051]	−0.142	[−0.181, −0.105]	0.050	[−0.018, 0.119]
Profil2 vs. Profile 5	−0.046	[−0.070, −0.026]	−0.157	[−0.198, −0.116]	0.111	[0.054, 0.165]
Profil3 vs. Profile 5	0.056	[0.031, 0.082]	0.036	[0.022, 0.053]	0.020	[−0.015, 0.051]
Profil4 vs. Profile 5	0.069	[0.038, 0.102]	0.097	[0.067, 0.131]	−0.028	[−0.081, 0.022]

Note. Profile 5 (High-Reactive-Balanced) was the reference group. LPA1–LPA4 correspond to Goal-Oriented, Low-Reactive, Moderate-Conflict, and High-Conflict profiles, respectively. For IGDS9-SF (continuous), estimates are unstandardized indirect effects; for smoking behavior (binary), estimates are logit-scale indirect effects. The contrast column represents the GAD-7-specific indirect effect minus the PHQ-9-specific indirect effect. 95% CIs are bias-corrected bootstrap confidence intervals based on 5000 draws. All models controlled for age, gender, household registration, university type, and grade. 95% CIs excluding zero indicate that the corresponding effect or contrast is statistically significant. IGDS9-SF = Internet Gaming Disorder Scale-Short-Form; GAD-7 = anxiety; PHQ-9 = depression.

**Table 8:** Full path coefficient.

Path	Estimate	SE	95% CI	*p*
a: paths: Profile → GAD-7				
Profile 1 vs. Profile 5	−2.759	0.096	[−2.942, −2.570]	<0.001
Profile 2 vs. Profile 5	−1.384	0.135	[−1.650, −1.120]	<0.001
Profile 3 vs. Profile 5	1.672	0.116	[1.450, 1.908]	<0.001
Profile 4 vs. Profile 5	2.054	0.165	[1.732, 2.379]	<0.001
a: paths: Profile → PHQ-9				
Profile 1 vs. Profile 5	−2.991	0.133	[−3.252, −2.730]	<0.001
Profile 2 vs. Profile 5	−3.294	0.112	[−3.506, −3.064]	<0.001
Profile 3 vs. Profile 5	0.757	0.130	[0.498, 1.009]	<0.001
Profile 4 vs. Profile 5	2.035	0.202	[1.630, 2.432]	<0.001
b: paths: GAD-7 → Outcome				
GAD-7 →problematic gaming	0.864	0.019	[0.827, 0.900]	<0.001
GAD-7 → Smoking (logit)	0.033	0.007	[0.019, 0.048]	<0.001
b: paths: PHQ-9 → Outcome				
PHQ-9 →problematic gaming	0.037	0.015	[0.007, 0.067]	0.015
PHQ-9 → Smoking (logit)	0.048	0.006	[0.035, 0.060]	<0.001
Direct paths: Profile → problematic gaming				
Profile 1 vs. Profile 5	−2.639	0.217	[−3.065, −2.211]	<0.001
Profile 2 vs. Profile 5	−3.652	0.173	[−3.975, −3.295]	<0.001
Profile 3 vs. Profile 5	−0.101	0.142	[−0.387, 0.169]	0.474
Profile 4 vs. Profile 5	−0.461	0.210	[−0.883, −0.054]	0.029
Direct paths: Profile → Smoking (logit)				
Profile 1 vs. Profile 5	0.572	0.130	[0.308, 0.813]	<0.001
Profile 2 vs. Profile 5	0.790	0.092	[0.610, 0.971]	<0.001
Profile 3 vs. Profile 5	0.280	0.070	[0.141, 0.414]	<0.001
Profile 4 vs. Profile 5	0.082	0.096	[−0.107, 0.263]	0.394
Total effects: Profile → problematic gaming				
Total 1 vs. 5 → problematic gaming	−5.134	0.220	[−5.554, −4.693]	<0.001
Total 2 vs. 5 → problematic gaming	−4.970	0.231	[−5.393, −4.502]	<0.001
Total 3 vs. 5 → problematic gaming	1.372	0.176	[1.031, 1.723]	<0.001
Total 4 vs. 5 → problematic gaming	1.389	0.258	[0.879, 1.895]	<0.001
Total effects: Profile → Smoking (logit)				
Total 1 vs. 5 → Smoking	0.337	0.130	[0.077, 0.582]	0.009
Total 2 vs. 5 → Smoking	0.587	0.091	[0.410, 0.766]	<0.001
Total 3 vs. 5 → Smoking	0.372	0.070	[0.234, 0.506]	<0.001
Total 4 vs. 5 → Smoking	0.248	0.096	[0.056, 0.430]	0.010

Note. GAD-7 = anxiety; PHQ-9 = depression. For problematic gaming, estimates are unstandardized. For smoking, estimates are logit-scale. All models controlled for age, gender, household registration, university type, and grade.

**Table 9:** Moderation analysis of demographic variables.

Type	Findings			
Dependent variable: IGDS9-SF				
Model Type	Model Explanatory Power (Total R^2^)	R^2^ Change (ΔR^2^)	Interaction Term Significance	Key Findings
Main Effects Model	0.103	—	—	LPA1–LPA4, gender, household registration type, institution type, and age significantly predicted problematic gaming
Gender × LPAs	0.106	0.003 (*p* < 0.001)	Significant	The interaction between LPA1–LPA4 and gender significantly increased explanatory power, indicating that gender moderates the relationship between LPA profiles and problematic gaming
Residence × LPAs	0.105	0.002 (*p* = 0.648)	Not Significant	The interaction between LPA1–LPA4 and household registration type did not increase explanatory power; the moderating effect of household registration type was not significant
Type of university × LPAs	0.103	0(*p* = 0.466)	Not Significant	The interaction between LPA1–LPA4 and institution type did not increase explanatory power; the moderating effect of institution type was not significant
Age × LPAs	0.105	0.002 (*p* < 0.001)	Significant	The interaction between LPA1–LPA4 and age significantly increased explanatory power, indicating that age moderates the relationship between LPA profiles and problematic gaming
Dependent variable: Smoker/Non-smoker				
Model Type	Model Fit (Nagelkerke R^2^)	Model Improvement (Δχ^2^, *p*)	Interaction Term Significance	Key Findings
Main Effects Model	0.131	—	—	LPA1-LPA4, gender, household registration type, institution type, and age significantly predicted smoking behavior
Gender × LPAs	0.134	Δχ^2^(4) = 24.890, *p* < 0.001	Significant	The interactions between LPA1–LPA3 and gender were significant, indicating that gender moderates the relationship between LPA profiles and smoking behavior
Residence × LPAs	0.131	Δχ^2^(4) = 1.504, *p* = 0.826	Not Significant	The interactions between LPA1–LPA4 and household registration type were not significant; the moderating effect of household registration type was not significant
Type of university × LPAs	0.132	Δχ^2^(4) = 8.959, *p* = 0.062	Not Significant	The overall moderation effect was not significant (*p* = 0.062), but the interaction between LPA3 and institution type was significant (OR = 0.753, *p* = 0.047)
Age × LPAs	0.131	Δχ^2^(4) = 3.355, *p* = 0.500	Not Significant	The interactions between LPA1-LPA4 and age were not significant; the moderating effect of age was not significant.

Note: With Profile 5 as the reference category. The main effects model controlled for gender, household registration type, institution type, and age. Note. Because these analyses are based on cross-sectional data, the indirect associations reported here should not be interpreted as causal mediation. The temporal order of variables is assumed for statistical modeling purposes but has not been empirically established. Findings are therefore hypothesis-generating and require replication in longitudinal designs.

## Data Availability

Data is unavailable due to privacy or ethical restrictions. The data underlying this article will be shared on reasonable request to the corresponding author.

## Supplementary Materials

The following supporting information can be downloaded at: https://www.mdpi.com/article/10.3390/bs16101958/s1, Table S1: Average posterior probabilities for the 6-class latent profile model (row = actual class, column = predicted class); Table S2: Comparison of latent profile characteristics (6-class model); Figure S1: Mean score patterns of the six latent profiles.

## Abbreviations

The following abbreviations are used in this manuscript:

BIS	Behavioral Inhibition System
BAS	Behavioral Activation System
DMSC-s	Dual-Mode of Self-Control Scale
GAD-7	Generalized Anxiety Disorder-7
IGDS9-SF	Internet Gaming Disorder Scale-Short-Form
PHQ-9	Patient Health Questionnaire-9

## References

[B1-behavsci-16-01958] Braddock K. H., Dillard J. P., Voigt D. C., Stephenson M. T., Sopory P., Anderson J. W. (2011). Impulsivity partially mediates the relationship between BIS/BAS and risky health behaviors. Journal of Personality.

[B2-behavsci-16-01958] Buono F. D., Paul E., Sprong M. E., Smith E. C., Garakani A., Griffiths M. D. (2020). Gaming and gaming disorder: A mediation model gender, salience, age of gaming onset, and time spent gaming. Cyberpsychology, Behavior, and Social Networking.

[B3-behavsci-16-01958] Carver C. S., White T. L. (1994). Behavioral inhibition, behavioral activation, and affective responses to impending reward and punishment: The BIS/BAS scales. Journal of Personality and Social Psychology.

[B4-behavsci-16-01958] Costes J. M., Bonnaire C. (2022). Spending money in free-to-play games: Sociodemographic characteristics, motives, impulsivity and internet gaming disorder specificities. International Journal of Environmental Research and Public Health.

[B5-behavsci-16-01958] Dvorak R. D., Simons J. S. (2009). Moderation of resource depletion in the self-control strength model: Differing effects of two modes of self-control. Personality and Social Psychology Bulletin.

[B6-behavsci-16-01958] Fluharty M., Taylor A. E., Grabski M., Munafò M. R. (2017). The association of cigarette smoking with depression and anxiety: A systematic review. Nicotine & Tobacco Research.

[B7-behavsci-16-01958] Garfield J. B., Lubman D. I., Yücel M. (2014). Anhedonia in substance use disorders: A systematic review of its nature, course and clinical correlates. Australian & New Zealand Journal of Psychiatry.

[B8-behavsci-16-01958] Gilbert K., Barch D., Luby J. (2020). The Overcontrol in Youth Checklist (OCYC): Behavioral and neural validation of a parent-report of child overcontrol in early childhood. Child Psychiatry & Human Development.

[B9-behavsci-16-01958] He Q., Li Z., Yang H. (2025). Dual-system perspectives: A meta-analytic comparison of striatal and prefrontal cortex activation patterns in substance addiction versus behavioral addiction. Acta Psychologica Sinica.

[B10-behavsci-16-01958] Hofmann W., Friese M., Strack F. (2009). Impulse and self-control from a dual-systems perspective. Perspectives on Psychological Science.

[B11-behavsci-16-01958] Hu F., Tai Z., Liu J. (2025). The positive effect of video-game play on college students’ anxiety and depression symptoms during the COVID-19 pandemic shelter-in-place lockdowns: Mixed methods study. JMIR Serious Games.

[B12-behavsci-16-01958] Kasza K. A., Edwards K. C., Tang Z., Stanton C. A., Sharma E., Halenar M. J., Taylor K. A., Donaldson E., Hull L. C., Day H., Bansal-Travers M., Limpert J., Zandberg I., Gardner L. D., Hammad H. T., Borek N., Kimmel H. L., Compton W. M., Hyland A. (2020). Correlates of tobacco product initiation among youth and adults in the USA: Findings from the PATH Study Waves 1-3 (2013-2016). Tob Control.

[B13-behavsci-16-01958] Khan A., Zeb I., Fang S. (2026). Navigating stigma and support: A qualitative study of help-seeking for mental health challenges among Chinese university students. International Journal of Qualitative Studies on Health and Well-Being.

[B14-behavsci-16-01958] Khantzian E. J. (1997). The self-medication hypothesis of substance use disorders: A reconsideration and recent applications. Harvard Review of Psychiatry.

[B15-behavsci-16-01958] Kim D. J., Kim K., Lee H. W., Hong J. P., Cho M. J., Fava M., Mischoulon D., Heo J. Y., Jeon H. J. (2017). Internet game addiction, depression, and escape from negative emotions in adulthood: A nationwide community sample of Korea. The Journal of Nervous and Mental Disease.

[B16-behavsci-16-01958] Kim Y., Jeong J. E., Cho H., Jung D. J., Kwak M., Rho M. J., Yu H., Kim D. J., Choi I. Y. (2016). personality factors predicting smartphone addiction predisposition: Behavioral inhibition and activation systems, impulsivity, and self-control. PLoS ONE.

[B17-behavsci-16-01958] Kim-Spoon J., Deater-Deckard K., Holmes C., Lee J., Chiu P., King-Casas B. (2016). Behavioral and neural inhibitory control moderates the effects of reward sensitivity on adolescent substance use. Neuropsychologia.

[B18-behavsci-16-01958] Koob G. F. (2021). Drug addiction: Hyperkatifeia/negative reinforcement as a framework for medications development. Pharmacological Reviews.

[B19-behavsci-16-01958] Kroenke K., Spitzer R. L., Williams J. B. (2001). The PHQ-9: Validity of a brief depression severity measure. Journal of General Internal Medicine.

[B20-behavsci-16-01958] Li Y. Z., Zhang Y., Jiang Y., Li H., Mi S., Yi G. J., Gu H. Y., Jiang Y. (2008). Reliability and validity of the Chinese version of the Behavioral Inhibition/Activation System Scale. Chinese Mental Health Journal.

[B21-behavsci-16-01958] Lindpere V., Winickoff J. P., Khan A. S., Dong J., Michaud T. L., Liu J., Dai H. D. (2023). Reasons for e-cigarette use, vaping patterns, and cessation behaviors among US adolescents. Nicotine & Tobacco Research.

[B22-behavsci-16-01958] Pontes H. M., Griffiths M. D. (2015). Measuring DSM-5 internet gaming disorder: Development and validation of a short psychometric scale. Computers in Human Behavior.

[B23-behavsci-16-01958] Qin L., Cheng L., Hu M., Liu Q., Tong J., Hao W., Luo T., Liao Y. (2020). Clarification of the Cut-off Score for Nine-Item Internet Gaming Disorder Scale–Short Form (IGDS9-SF) in a Chinese context. Frontiers in Psychiatry.

[B24-behavsci-16-01958] Schou Andreassen C., Billieux J., Griffiths M. D., Kuss D. J., Demetrovics Z., Mazzoni E., Pallesen S. (2016). The relationship between addictive use of social media and video games and symptoms of psychiatric disorders: A large-scale cross-sectional study. Psychology of Addictive Behaviors.

[B25-behavsci-16-01958] Sheer V. C., Mao C. (2017). Cigarette initiation among Chinese male teenagers in early smoking interactions. Health Communication.

[B26-behavsci-16-01958] Song H., Yang X., Yang W., Dai Y., Duan K., Jiang X., Huang G., Li M., Zhong G., Liu P., Chen J. (2023). Cigarettes smoking and e-cigarettes using among university students: A cross-section survey in Guangzhou, China, 2021. BMC Public Health.

[B27-behavsci-16-01958] Spitzer R. L., Kroenke K., Williams J. B., Löwe B. (2006). A brief measure for assessing generalized anxiety disorder: The GAD-7. Archives of Internal Medicine.

[B28-behavsci-16-01958] Sussman S., Sinclair D. L. (2025). Substance and behavioral addictions-Two sides of the same coin: Commentary on Grubbs and Boness (2025). Journal of Psychopathology and Clinical Science.

[B29-behavsci-16-01958] van den Brink W. (2017). ICD-11 Gaming Disorder: Needed and just in time or dangerous and much too early?. Journal of Behavioral Addictions.

[B30-behavsci-16-01958] Wang W., Bian Q., Zhao Y., Li X., Wang W., Du J., Zhang G., Zhou Q., Zhao M. (2014). Reliability and validity of the Chinese version of the Patient Health Questionnaire (PHQ-9) in the general population. General Hospital Psychiatry.

[B31-behavsci-16-01958] Wang Y., Chen R., Zhang L. (2018). Reliability and validity of the Generalized anxiety disorder-7 in Chinese general hospital inpatients. Journal of Clinical Psychiatry.

[B32-behavsci-16-01958] Wu N., Ding R., Yang X. (2025). Comparative analysis of the diffusion trends of e-cigarette use and cigarette smoking among school students in China: A cross-sectional study. Zhongguo Gonggong Weisheng.

[B33-behavsci-16-01958] Xie D. J., Wang L. G., Tao T., Fan C. L., Gao W. B. (2014). Validity and reliability of the Chinese version of the dual-mode of self-control scale for adolescents. Chinese Mental Health Journal.

[B34-behavsci-16-01958] Xue J., Li Z., Zhang W., Li W., Liu L., Zhang Z. (2023). The longitudinal impact of reinforcement sensitivity on internet addiction among college students: The mediating role of self-control. Frontiers in Psychiatry.

[B35-behavsci-16-01958] Zeng X., Han X., Zheng D., Jiang P., Yuan Z. (2024). Similarity and difference in large-scale functional network alternations between behavioral addictions and substance use disorder: A comparative meta-analysis. Psychological Medicine.

[B36-behavsci-16-01958] Zhou L., Zhang Y., Shadel W. G. (2023). Impact of social norms on Chinese college students’ tobacco use. Current Psychology.

