# Supplementary material for: Latent Profiles of Motivational and Regulatory Systems and Their Differential Associations with Problematic Gaming and Smoking Behavior Among Chinese College Students: A Cross-Sectional Study
Source: Behav Sci (Basel). 2026 Oct 21;16(10):1958. doi: 10.3390/bs16101958 (PMC13701243; doi:10.3390/bs16101958)
Supplement: Supplementary file 1 [file behavsci-16-01958-s001.zip › behavsci-4570696-supplementary.pdf]

In the six-class latent profile analysis, model estimation terminated normally, with 33 free parameters; the H0 log-likelihood was -160405.160, the MLR scaling correction factor was 1.3523, AIC = 320876.320, BIC = 321123.461, sample-size adjusted BIC = 321018.590, and entropy = 0.909. Based on the estimated model, the proportions for the six profiles were C1 = 4.75% (n ≈ 627.51), C2 = 2.03% (n ≈ 268.13), C3 = 16.22% (n ≈ 2143.16), C4 = 5.98% (n ≈ 790.28), C5 = 60.89% (n ≈ 8046.37), and C6 = 10.14% (n ≈ 1339.55), whereas the proportions based on their most likely latent class membership were 4.44%, 2.00%, 16.19%, 5.96%, 61.67%, and 9.75%, respectively. Classification information showed that the diagonal of the average posterior probability matrix was 0.841, 0.893, 0.906, 0.979, 0.950, and 0.904, and the diagonal of the classification probabilities for the most likely latent class membership was 0.787, 0.880, 0.905, 0.977, 0.962, and 0.869, indicating good classification quality. For model comparison, the VLMR-LRT (5 vs. 6 classes) had 2×ΔLL = 1301.603, Δdf = 5, $p < 0.001$, and the LMR adjusted LRT was 1274.735, $p < 0.001$; however, the TECH14 parametric bootstrap LRT had an approximate p = 0.0000 with only 5 successful bootstrap draws, and 3 of these draws had both a smaller LRT value and a non-replicated best log-likelihood for the 6-class model, so this p-value may not be trustworthy because of local maxima, and it is recommended that LRTSTARTS be used to increase random starts. In addition, the MODEL command warning stated that "All variables are uncorrelated with all other variables within class," so it is necessary to confirm whether this is consistent with the analysis specification; the best log-likelihood has been replicated, but the output recommends re-running with at least twice the random starts to verify that the best log-likelihood is still obtained and replicated. The detailed results can be found in Tables 1 and 2, as well as in Figure 1.

Table S1. Average posterior probabilities for the 6-class latent profile model (row = actual class, column = predicted class).

	C1	C2	C3	C4	C5	C6
C1	0.841	0.002	0.065	0.000	0.092	0.000
C2	0.002	0.893	0.061	0.044	0.000	0.000
C3	0.023	0.009	0.906	0.003	0.059	0.000
C4	0.000	0.015	0.005	0.979	0.000	0.000
C5	0.010	0.000	0.018	0.000	0.950	0.021
C6	0.000	0.000	0.000	0.000	0.096	0.904

Table S2. Comparison of latent profile characteristics (6-class model).

Class	BAS	BIS	Control System (KZ)	Impulse System (CD)	Proportion	Profile Characteristic Description
C1	39.357	9.853	36.313	20.453	4.75%	High activation + High control, Low inhibition + Low impulse
C2	17.977	6.311	30.400	22.125	2.03%	Low activation + Moderate

						control,Low inhibition
C3	28.971	10.782	26.172	28.850	16.22%	Moderate and balanced
C4	14.523	5.455	10.696	13.324	5.98%	Low across all systems
C5	38.900	14.757	30.908	30.456	60.89%	Relatively high and balanced
C6	46.255	18.599	34.357	34.790	10.14%	High across all systems

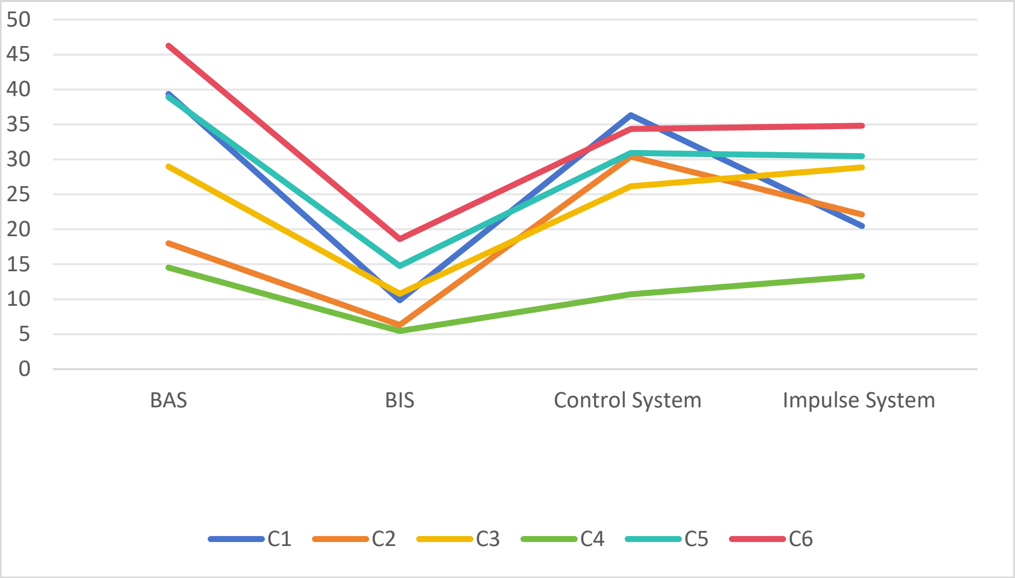

Figure S1. Mean score patterns of the six latent profiles.
